# Occurrence and reasons for on-farm emergency slaughter of cattle in Norway

**DOI:** 10.3389/fvets.2022.1067489

**Published:** 2022-12-01

**Authors:** Gíslína Skúladóttir, Ingrid Hunter-Holmøy, Clare Joan Phythian, Guro Myhrene, Adam Dunstan Martin

**Affiliations:** ^1^Department of Production Animal Clinical Sciences, Faculty of Veterinary Medicine, Norwegian University of Life Sciences, Ås, Norway; ^2^Section for Small Ruminant Research, Faculty of Veterinary Medicine, Norwegian University of Life Sciences, Sandnes, Norway; ^3^Norwegian Food Safety Authority, Sandnes, Norway

**Keywords:** OFES, cattle, animal welfare, food safety, cattle mortality, slaughter, public health, sustainable cattle raising

## Abstract

On-farm emergency slaughter (OFES) accounts for more than 4% of all cattle slaughter in Norway. The practice raises questions about animal welfare, public health, and the sustainability of cattle production. The objective of this study was to describe the reasons for OFES as stated on the OFES veterinary certificate. Veterinary certificates for OFES for each animal slaughtered in four chosen slaughterhouses from 4 months (January–April–July–October) in 2018 were transcribed into a database. Secondary data were extracted from national cattle databases and used to supplement primary data with information on breed, sex, and birth date. Breeds were divided into dairy and beef cattle. The reasons for slaughter were reported in text on the certificates and were categorized in the study into 5 reasons: recumbency, mammary gland, obstetrics, locomotion, and other, with a total of 20 subcategories for detail. In total, 2,229 forms were included in the study. Thirteen breeds were represented, although dominated by Norwegian Red within dairy and crossbreed within beef. Of the cattle in the study, 46% were slaughtered for locomotion reasons, thereof almost half for lameness. Furthermore, 23% of the cattle in the study were slaughtered for recumbency and 17% for prolapse or dystocia. A higher proportion of dairy cows were slaughtered because of reasons related to mammary glands than beef cows, 10 and 2%, respectively. Almost 30% of beef cows were slaughtered for obstetrics reasons compared to 12% of dairy cows. The results of this study shed light on the reasons for OFES, which is highly relevant to greater discussions of sustainability in cattle production and animal welfare related to on-farm mortality.

## Introduction

On-farm mortality and planned culling are the main end-of-life events for production animals. On-farm mortality is an unexpected event encompassing unassisted death, euthanasia, or on-farm emergency slaughter (OFES). This differs from culling, where a planned decision was made to remove the animal from the herd, either through sale or slaughter (1). An acutely injured animal may be euthanized or undergo OFES, or be casualty slaughtered (at a slaughterhouse). Per the European Union (EU) regulation (EC) No 1/2005, rules concerning the state of an animal before and during transport to slaughter are getting stricter, and thus casualty slaughter is often not a viable option anymore (2). After rearing an animal, it can become an economical burden on the farmer if it is lost unexpectedly; length of its productive life is shortened, as the animal has not lived its expected lifespan. This applies especially if the animal needs to be euthanized, whilst OFES may salvage some of the value of the compromised animal and therefore limit the loss. On-farm emergency slaughter is legal in the EU, the countries of the European Economic Area (EEA), the United Kingdom and some jurisdictions in Canada (3). Nevertheless, some European countries do not practice OFES and in the remaining EU countries, it is used limitedly because many slaughterhouses do not offer the option of OFES (3–6). Conversely, Australia, New Zealand and the United States of America do not allow for OFES (3, 7).

The prerequisites for OFES of cattle in the EU are; the animal must have had a recent accident or unforeseeable incident, have an unaffected general condition, and be ineligible for transport (8). As Norway is not in the EU, but a part of the EEA, the EU regulations are later committed to the Norwegian legislation, with the option of provisions special to Norway, if appropriate. The Norwegian Food Safety Authority has published guidelines, interpreting the legislation, to harmonize the practice of OFES in Norway (9). These guidelines have been changed 2 since the start of this study, in March 2021, and September 2022, while the legislation has remained unchanged. As the legislation has been interpreted in 3 different ways in Norway, different implantation of the legislation might affect the difference between the practice of OFES in varying countries (3). That difference is clearest in the contrast between the proportion of OFES of cattle of all cattle slaughter in the different reported countries, with Norway being 4.2% in 2018 (10), while the Republic of Ireland, Northern Ireland, and the Netherlands have reported 0.01, 0.11, and 0.90%, respectively (5).

The perception of veterinarians on the use of OFES is conflicted, between animal welfare and/or public health concerns, and the economic interests of the farmer (6, 11, 12). The public health concern is based on the possibility of infections carried with the animal to the consumer by way of poorer slaughter hygiene (13). Casualty slaughtered cattle have been shown to have a wider range of anthelmintic drug residues in the muscle of those than cattle conventionally slaughtered in a slaughterhouse (14). This could be because of insufficient food-chain information, or that the information is not logged, and then forgotten as the slaughter of the animal had not been planned when the animal was given anthelmintic drugs. The animal welfare concerns relate to both the reason for slaughter, as well as the wait time from certification to slaughter, as the animal may be suffering while waiting. Neither aspect has been researched well, and there is little data available to conclude on these concerns. The decision process is often quite complex, both for farmer, veterinarian, and slaughterman, and could possibly be helped with good guidelines and decision trees, as discussed in research from both British Columbia/Canada and the Republic of Ireland (6, 12).

Previously, there has only been one study published on the reasons and use of OFES (7). That study describes the situation in British Colombia, Canada and is not directly comparable to Europe, as the legal framework is different (4, 7). The frequency of OFES in Norway compared to the frequency reported in other countries also means further knowledge of OFES is required. Therefore, the objective of this article is to describe the reasons provided on veterinary certificates for the OFES of cattle in Norway.

## Methods and materials

### Study population

The three largest cattle slaughterhouses in Norway, identified using data published by Animalia – The Norwegian Meat Research Center (10) were included. Additionally, one private slaughterhouse was selected to be included in the study based on its location in a highly cattle-dense area. Veterinary certificates for OFES from the first month in each quarter (January, April, July and October) of 2018 were selected to be included in the study. All slaughterhouses gave access to their numbers of total cattle slaughtered in 2018 as well as the total number of OFES processed in 2018.

### Data sources

#### On-farm emergency slaughter

The veterinary certificates for OFES are collected and stored by the Norwegian Food Safety Authority in each slaughterhouse. The Norwegian Food Safety Authority granted access to the physical copies of the handwritten veterinary certificates. The veterinary certificates contain identification information about the farmer and animal, the reason for OFES, drug history for the animal for the last 30 days, including regulated withdrawal times and the signatures from the veterinarian, farmer, and registered slaughterman. Cattle born in Norway are to be marked by an ear tag including an identification number for the farm they were born to (8 digits) and the animals' own id (4 digits) (15). The first author entered all legible data from the OFES veterinary certificate into Microsoft Access 365 database. All illegible data as well as data not recorded on the certificates were entered as missing in the database.

The reasons for OFES were determined based on text from the veterinary certificates for OFES. The reasons for slaughter were then categorized into 20 categories that were grouped into 5 categories: recumbency, mammary gland, obstetrics, locomotion, and other. Criteria for categories and subcategories are shown in Table 1. The category of locomotion encompasses all reasons that were connected to the limbs of the animal which were likely to result in an abnormality of gait, including “lame” animals, acute soft tissue trauma to legs, fractures of legs and arthritis. In cases where multiple reasons for OFES were listed by the attending veterinarian, one category was chosen based on what the authors interpreted as the primary reason for OFES. Data collection was performed in the last quarter of 2019.

**Table 1:** Inclusion criteria for categories of reason for OFES, including the sorting of subcategories to categories.

**Category**	**Subcategory**	**Inclusion criteria for category**
Recumbency		
	Unable to stand	Cases unable to stand, but unknown cause
	Milk fever	Cases of milk fever, not recovering
	Splits	Have done the splits, recumbent.
	Palsy	Cases of muscle, nerve or tendon damage.
Mammary Gland		
	Mastitis	Cases of mastitis
	Udder Damage	Cases of trauma to the udder as well as risk to mastitis
Obstetrics		
	Prolapse	Cases with a current vaginal or uterine prolapse, sometimes in combination with a rectal prolapse
	Dystocia	All reasons relating to the upcoming calving or just calved. Cases of calving difficulties, uterine torsion
Locomotion		
	Lame	Cases of lame animals
	Acute Leg Trauma	Cases of acute soft tissue trauma to the legs, thus excluding fractures
	Fracture	Cases of fractures or tentative factures
	Arthritis	Cases of arthritis
Other		
	Trauma	Cases of trauma (not to legs or udder)
	Internal	Cases of clinical signs of internal cause
	Poor Appetite	Cases reported having poor appetite
	Wild	Including animals that can't be caught after being released outside, as well as aggressive and uncontrollable individuals
	Illegible	The reason for slaughter was illegible on the certificate to all authors of the paper
	Management	Cases where no medical reason was stated, only that the farmer wished for OFES
	Empty	Cases where no reason included on the certificate
	Rectal Prolapse	Cases of rectal prolapse, not in combination with other prolapse

#### Cattle databases

The unique animal ID obtained from the OFES veterinary certificate was used to extract secondary data from the voluntary nationwide recording systems for cattle farming. These are the Norwegian Dairy Herd Recording System (NDHRS) and the Norwegian Beef Cattle Recording System (NBCRS). In 2018, 98 % of Norwegian dairy herds were enrolled in the NDHRS and 70% of beef herds in the NBCRS (16, 17). The recording systems contain information on cow pedigree, and the production and health of individual animals in enrolled herds. Information on birth date, slaughter date, sex, breed, and slaughter classification were extracted from the NDHRS/NBCRS to supplement the primary data. Additionally, parity and the most recent calving date were extracted when applicable. Individuals not successfully matched in the initial extraction were examined for transcription mistakes, and information was extracted for additionally identified individuals.

### Data management

Further data management and analysis were performed using Stata SE/15 (Stata Corp., College Station, TX, USA). Data were checked for duplicates and transcribing errors were corrected. The primary dataset was merged with the supplementary data from the voluntary nationwide recording systems in Stata. Animals of the following breeds were classified as dairy; Norwegian Red, Jersey, Trønder and Nordlands, Brown Swiss, Holstein, Milk Simmental, and Raukolle while crossbreeds, Charolais, Limousine, Hereford, Aberdeen Angus, and Beef Simmental were classified as beef cattle. This gave two groups of animal production systems. Animals with no data on breed were not included in the analysis by production system.

The age of the animal in days was calculated by subtracting the birthdate from the slaughter date. Age was used to group animals into five animal type categories similar to that used for slaughter classification (18). Calf was any animal, both female and male, 300 days old and younger, bull is a male over 301 days old, heifer is a female from 301 to 760 days old. Young cow is a female from 761 to 1,460 days old and cow is a female older than 1,460 days old.

### Descriptive statistics

Frequency distributions were used to describe categorical data. Total numbers and percentages were extracted for all records, and each production system for the variables slaughterhouse, slaughter month, sex, and animal type. The reasons for OFES were tabulated by production system and total numbers and percentages, as well as tabulated for each production system by animal type.

## Results

A total of 2,247 cases were recorded from the four slaughterhouses. Of those, 18 were from a month outside the study period, because they were sorted by *postmortem* inspection date, not OFES date. Thus, 2,229 cases were included in the database for analysis, but 32 of those veterinary certificates did not include a complete 12-digit animal ID, making it impossible to request secondary data from the voluntary national cattle databases. Further, 451 could not be matched to any animals in the cattle databases, leaving 1,746 with secondary data. Of those, 1,563 included breed information, used to sort into two different production systems.

Slaughterhouses A, B, C and D reported that 5, 4, 4, and 3% of their total cattle slaughter in 2018 was OFES, respectively. The cases collected accounted for 30, 27, 34, and 38% of the OFES records in each respective slaughterhouse in 2018. Table 2 shows the distribution of the total number of cases and tabulates them by production system for each slaughterhouse, slaughter month, sex, and animal type. Half of the study, 53% were dairy cattle, of which, Norwegian Red was the most common breed of dairy cattle breeds in the study sample (73% of dairy cattle). Meanwhile, 17% of the whole study sample were beef cattle breeds where crossbreeds were the most common breed, accounting for 58% of beef cattle. However, 30% (*n* = 666) of the whole study sample was missing information on breed (and production system). The division of sex within the dairy production system was about 20–80% male-female, respectively, while it was 30–70% within beef production system. Further, the beef production system had a more even distribution of animal types, 7, 25, 16, 26, 26% of calf, bull, heifer, young cow, and cow, respectively, while (adult) cows accounted for 43% of the OFES from dairy production systems. This follows the statistics from Animalia that 8% of all milking cows are slaughtered by undergoing OFES instead of conventional slaughter (10). OFES-cases of dairy cattle were evenly distributed throughout the sampled months (23–26%). OFES of beef cattle were more frequent in April and less frequent in January, accounting for 37 and 14% of the total sample, respectively.

**Table 2:** Descriptive table of database, showing variables by production system, missing and total.

	**Production system**			**All**
**Variable**	**Dairy**	**Beef**	**Missing**	**Total**
Abattoir				
A	91 (7.6%)	54 (14.5%)	130 (19.5%)	275 (12.3%)
B	211 (17.7%)	77 (20.7%)	205 (30.8%)	493 (22.1%)
C	580 (48.7%)	142 (38.2%)	198 (29.7%)	920 (41.3%)
D	309 (26%)	99 (26.6%)	133 (20.0%)	541 (24.3%)
Slaughter month				
January	305 (25.6%)	53 (14.2%)	145 (21.8%)	503 (22.6%)
April	270 (22.7%)	138 (37.1%)	198 (29.7%)	606 (27.2%)
July	307 (25.8%)	91 (24.5%)	179 (26.9%)	577 (25.9%)
October	309 (25.9%)	90 (24.2%)	144 (21.6%)	543 (24.3%)
Sex				
Male	235 (19.7%)	107 (28.8%)	144 (21.7%)	486 (21.8%)
Female	956 (80.3%)	265 (71.2%)	520 (78.3%)	1,741 (78.1%)
Missing	0 (0%)	0 (0%)	2 (0.003%)	2 (0.1%)
Animal type				
Calf	33 (2.7%)	25 (6.7%)	15 (2.3%)	73 (3.3%)
Bull	212 (17.8%)	91 (24.5%)	51 (7.7%)	354 (15.9%)
Heifer	150 (12.6%)	61 (16.4%)	28 (4.2%)	239 (10.7%)
Young cow	288 (24.2%)	97 (26.1%)	79 (11.9%)	464 (20.8%)
Cow	507 (42.6%)	98 (26.3%)	97 (14.6%)	702 (31.5%)
Missing	1 (0.1%)	0 (0%)	396 (59.5%)	397 (17.8%)
Total	1,191 (53.4%)	372 (16.7%)	666 (29.9%)	2,229 (100%)

**Figure 1:**
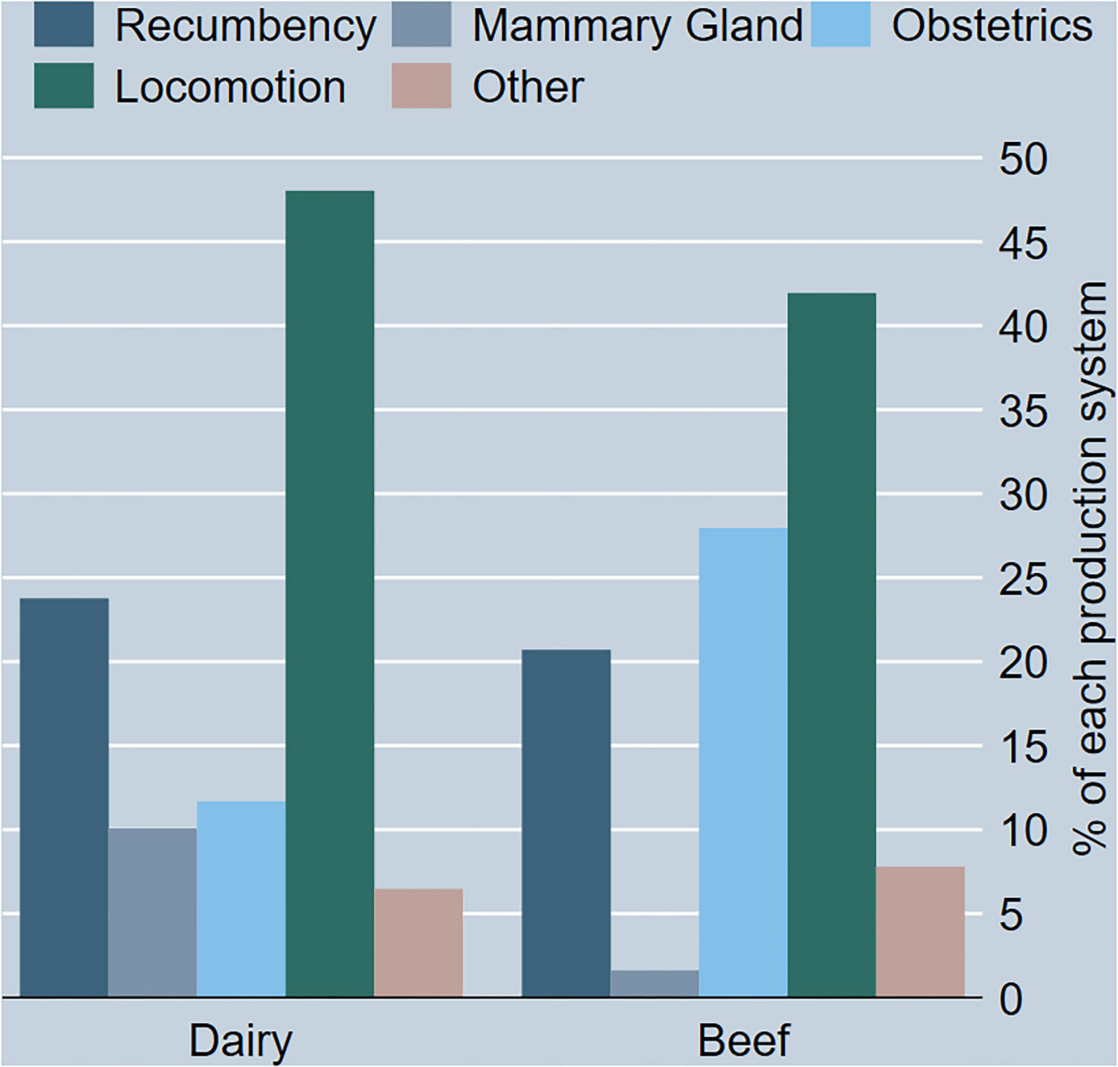
Bar graph showing the proportion of each reason for OFES for the two production systems *n* = 1,563.

Table 3 shows the occurrence of reasons for OFES by production system and for the total number of cases. Locomotory reasons account for 46% of total OFES in this study. Almost half of those (45%) were subcategorized as lame. Thus, lameness accounted for 21% of the total OFES in this study. Further 10% of dairy cattle suffered mammary gland issues before OFES, while only 2% of beef cattle did. Obstetrical reasons accounted for a larger proportion of the beef cattle certificates than the dairy cattle certificates 28 and 12%, respectively, see also Figure 1. One-fifth of the OFES were reportedly recumbent (23%) of which a majority were categorized as palsy. Trauma not related to locomotory, or mammary gland reasons accounted for only 3% of the total sample.

**Table 3:** Descriptive table showing number and percentage of total of each production system within each subcategory for OFES.

**Causes**	**Production system**		
	**Dairy**	**Beef**	**Total**
Recumbency			
Unable to stand	29 (2.4%)	9 (2.4%)	55 (2.5%)
Milk fever	25 (2.1%)	3 (0.8%)	38 (1.7%)
Splits	50 (4.2%)	18 (4.8%)	89 (4%)
Palsy	179 (15%)	47 (12.6%)	328 (14.7%)
Mammary gland			
Mastitis	21 (1.7%)	0 (0%)	29 (1.3%)
Udder damage	99 (8.3%)	6 (1.6%)	128 (5.7%)
Obstetrics			
Prolapse	56 (4.7%)	63 (17%)	201 (9%)
Dystocia	83 (7%)	41 (11%)	175 (7.8%)
Locomotion			
Lame	268 (22.5%)	62 (16.7%)	467 (21%)
Acute leg trauma	227 (19.1%)	68 (18.3%)	418 (18.7%)
Fracture	72 (6%)	25 (6.7%)	139 (6.2%)
Arthritis	5 (0.4%)	1 (0.3%)	8 (0.4%)
Other			
Trauma	30 (2.5%)	12 (3.2%)	63 (2.8%)
Internal	25 (2.1%)	7 (1.9%)	42 (1.9%)
Poor appetite	2 (0.2%)	0 (0%)	8 (0.4%)
Wild	8 (0.7%)	3 (0.8%)	16 (0.7%)
Illegible	1 (0.1%)	0 (0%)	4 (0.2%)
Management	8 (0.7%)	0 (0%)	10 (0.4%)
Empty	2 (0.2%)	0 (0%)	2 (0.1%)
Rectal prolapse	1 (0.1%)	7 (1.9%)	9 (0.4%)
Total	1,191 (53.4%)	372 (16.7%)	2,229 (100%)

Records missing data on production system are n = 666 (29.9%) included in the total.

Tables 4, 5 show the occurrence of reasons for OFES by animal type for production system dairy and beef, respectively. Of the beef heifers, almost half, 46% were OFES for obstetrical reasons, with only 17% of dairy heifers being OFES for the same reasons. For the younger animals, [calves, bulls and heifers, (most under 2 years of age)], fractures were more frequent than for older animals, 12–18% vs. 1–4%, respectively. When delving into different age brackets, it was clear that lameness continued to a bigger or equal proportion of the study sample as fractures, until going as young as 220 days. At that age, lameness dropped from around 25%, equal to fractures, to around 10%. The exception is the beef heifers, where only 8% of heifers had a fracture for OFES.

**Table 4:** Descriptive table showing number and percentage of total of each animal type within each subcategory of OFES, *n* = 1,190, only including animals from a dairy production system.

**Causes**	**Animal type**				
	**Calf**	**Bull**	**Heifer**	**Young cow**	**Cow**
Recumbency					
Unable to stand	-	2 (0.9%)	1 (0.7%)	6 (2.1%)	20 (3.9%)
Milk fever	-	-	-	-	25 (4.9%)
Splits	2 (6.1%)	-	7 (4.6%)	21 (7.3%)	20 (3.9%)
Palsy	2 (6.1%)	18 (8.5%)	24 (16%)	42 (14.6%)	93 (18.3%)
Mammary gland					
Mastitis	-	-	1 (0.7%)	7 (2.4%)	13 (2.6%)
Udder damage	-	-	3 (2%)	33 (11.5%)	63 (12.4%)
Obstetrics					
Prolapse	-	-	4 (2.7%)	25 (8.7%)	27 (5.3%)
Dystocia	-	-	22 (14.7)	36 (12.5%)	25 (4.9%)
Locomotion					
Lame	7 (21.2%)	87 (41.0%)	20 (13.3%)	47 (16.3%)	107 (21.2%)
Acute leg trauma	14 (42.4%)	61 (28.7%)	27 (18%)	45 (15.6%)	80 (15.8%)
Fracture	6 (18.2%)	29 (13.7%)	26 (17.3%)	5 (1.7%)	6 (1.2%)
Arthritis	-	3 (1.4%)	1 (0.7%)	-	1 (0.2%)
Other					
Trauma	1 (3%)	8 (3.8%)	6 (4%)	6 (2.1%)	9 (1.8%)
Internal	1 (3%)	1 (0.5%)	2 (1.3%)	9 (3.1%)	12 (2.4%)
Poor appetite	-	-	-	2 (0.7%)	-
Wild	-	1 (0.5%)	5 (3.3%)	2 (0.7%)	-
Illegible	-	1 (0.5%)	-	-	-
Management	-	-	1 (0.7%)	2 (0.7%)	4 (0.8%)
Empty	-	-	-	-	2 (0.4%)
Rectal prolapse	-	1 (0.5%)	-	-	-
Total = 1,190 (100%)	33 (2.7%)	212 (17.8%)	150 (12.6%)	288 (24.2%)	507 (42.6%)

**Table 5:** Descriptive table showing number and percentage of total of each animal type within each subcategory of OFES, *n* = 372, only including animals from a beef production system.

**Causes**	**Animal type**				
	**Calf**	**Bull**	**Heifer**	**Young cow**	**Cow**
Recumbency					
Unable to stand	-	1 (1.1%)	1 (1.6%)	3 (3.1%)	4 (4.1%)
Milk fever	-	-	-	-	3 (3.1%)
Splits	-	3 (3.3%)	5 (8.2%)	3 (3.1%)	7 (7.1%)
Palsy	4 (16%)	3 (3.3%)	10 (16.4%)	14 (14.4%)	16 (16.3%)
Mammary gland					
Mastitis	-	-	-	-	6 (6.1%)
Udder damage	-	-	-	-	
Obstetrics					
Prolapse	-	-	16 (26.2%)	27 (27.8%)	20 (20.4%)
Dystocia	-	-	12 (19.7%)	16 (16.5%)	13 (13.3%)
Locomotion					
Lame	4 (16%)	32 (35.1%)	2 (3.3%)	12 (12.4%)	12 (12.2%)
Acute leg trauma	7 (28%)	34 (37.4%)	7 (11.5%)	11 (11.3%)	9 (9.2%)
Fracture	4 (16%)	11 (12.1%)	5 (8.2%)	4 (4.1%)	1 (1%)
Arthritis	-	1 (1.1%)	-	-	-
Other					
Trauma	-	4 (4.4%)	3 (4.9%)	2 (2.1%)	3 (3.1%)
Internal	1 (4%)	1 (1.1%)	-	2 (2.1%)	3 (3.1%)
Poor appetite	-	-	-	-	-
Wild	1 (4%)	1 (1.1%)	-	1 (1%)	-
Illegible	-	-	-	-	-
Management	-	-	-	-	-
Empty	-	-	-	-	-
Rectal prolapse	4 (16%)	-	-	2 (2.1%)	1 (1%)
Total = 372 (100%)	25 (6.7%)	75 (20.2%)	61 (4.3%)	97 (26.1%)	98 (26.3%)

**Figure 2:**
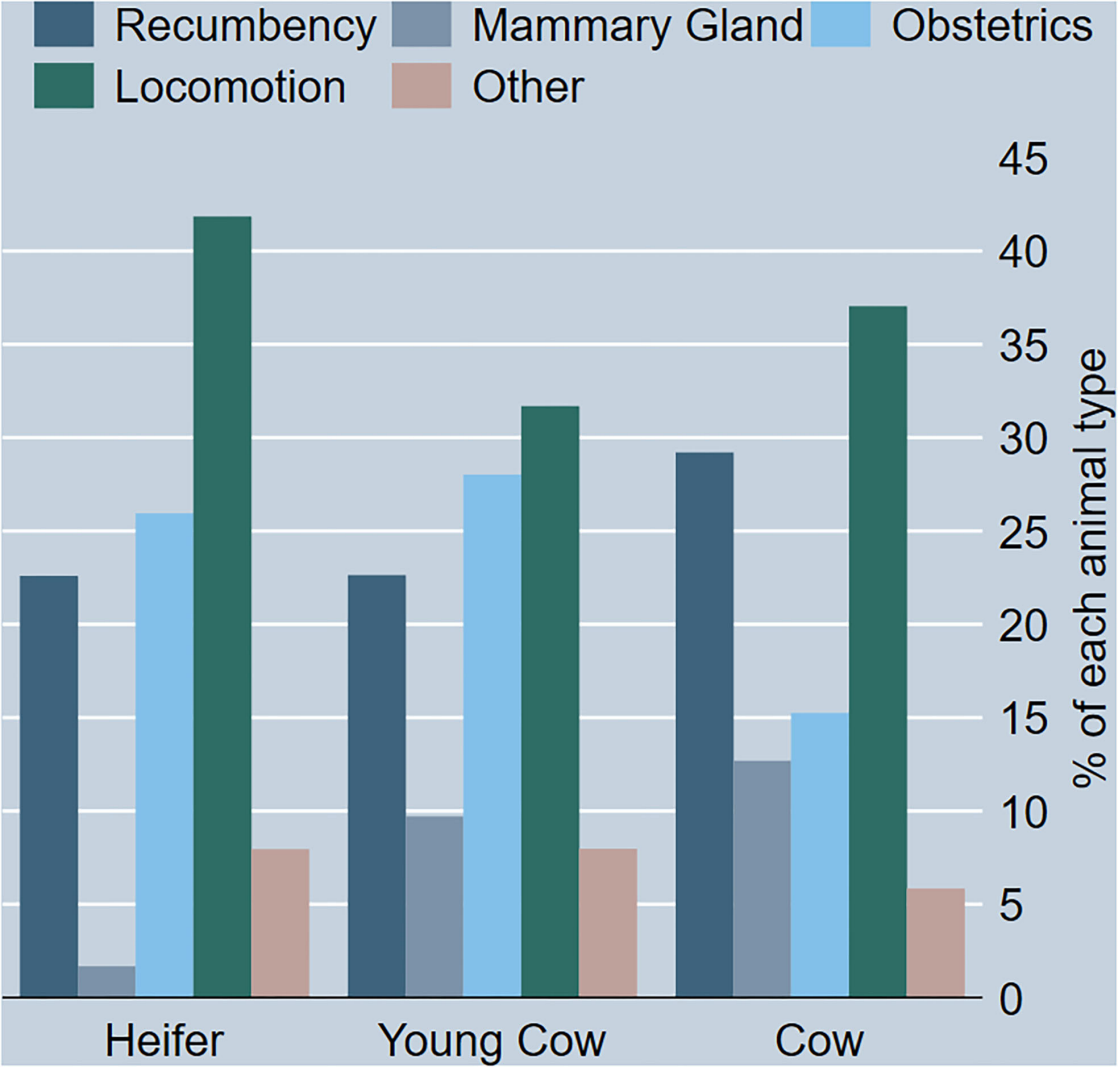
Bar graph showing the proportion of each reason for OFES for 3 animal types; Heifer, Young Cow and Cow *n* = 1,405.

The proportion of locomotion as the reason for OFES for heifers is larger than for the older age groups (young cow and cow; Figure 2). Furthermore, obstetrics as the reason for OFES were more frequent for heifers and young cows compared to cows.

## Discussion

This is the first study in Europe and the second in the world to focus on documenting the reasons for OFES (7). Almost half of all the cases reported locomotory reasons for OFES. Of the locomotory cases, half were subcategorized as lame. The current study found lameness the reason provided for OFES for 1 out of every 10 cases, in every age group, and over 20% in overall average. In contrast, a study performed in British Columbia/Canada found that only 9% of all the cattle underwent OFES because of lameness which the study points out is a chronic condition (7). The OFES guidelines in British Columbia clearly state that animals suffering from chronic conditions were not eligible for OFES, although no specific guidance on lame animals is offered (7). Therefore, OFES of lame cattle in Norway might explain some of the difference between the occurrence of OFES in Norway in contrast to other countries (5, 10).

Lameness is a cause of suffering and an area of animal welfare concern (1, 19, 20). The Norwegian guidelines for OFES in place in 2018 when this study was performed, specifically stated that lame animals were eligible for OFES, despite the condition not necessarily being acute or the result of an accident. In contrast, the current guidelines for OFES in Norway, updated in 2022, only allow for newly acute lame animals, within certain timeframes (9). While OFES may be the best option for an acutely lame animal it has not been mentioned as one of the biggest causes of on-farm mortality in Estonia (21). The reason for these apparent differences is unclear but could be caused by Estonia treating their lame animals, so they can continue their production, or if they are sent to conventional slaughter despite being ineligible for transport in some cases, or perhaps there are fewer lame cattle in Estonia. Further, locomotor disorders were found to be more common among euthanized cows than cows dying naturally in a Danish study, theorized to be because lameness is rarely the cause of natural death, but can be cause for euthanasia (22). Comparing the proportion of the whole between OFES and on-farm mortality might prove ill-advised, as certain categories, such as metabolic and digestive disorders only occur within on-farm mortality meaning the comparison would be skewed. None the less, lameness is a welfare problem that can be fixed with OFES for individual animals. The trade-off is that the ready-available option of OFES in Norway offers veterinarians and farmers an easy-out to rely on, instead of focusing on prevention and treatment of lameness.

The current study showed that the proportion of young animals (calves, bulls, and heifers) reported having a fracture which resulted in OFES being 12–18%, with beef heifers being intermediate (8%). This is in stark contrast to the proportions of fractures in older animals (1–4%). The outlier of beef heifers can possibly be explained by the big proportion, 46%, within that group that had obstetrical reasons for OFES. The proportions discussed in this article, within animal types are always of the whole of the representatives for that animal type in the study sample. Therefore, when discussing high proportions of specific causes within certain animal types that don't have cases of OFES for mammary gland reasons or obstetrical reasons, caution should be applied to prevent the overinterpretation of these data. In Norway, beef steers and replacement heifers are commonly housed in group pens, often on slatted floors. In these housing conditions, there is little shelter for each animal, which may lend itself to more traumas, caused by other animals or by simply slipping. By the end of 2021, 67% of dairy cows were kept in free-stalls and the rest in tie stalls, with mattresses in the lying areas (23). The topic of housing is a large part of the new animal welfare program on cattle in Norway (24) and the results of this study could stand as further argumentation for keeping young animals in pens with enough space and options for shelter. The difference in housing and the rate of growth, between the different animal groups, may, therefore, be an important factor in deciding which reasons for OFES are more common, but this would need further study to confirm.

Almost half of the beef heifers in this study, and one-fifth of other heifers and cows in this study underwent OFES for obstetrical reasons. Prolapse accounts for half of those, which has been seen to be more common in beef breeds, than dairy (25). Further, crossbreeds accounted for the majority of beef cattle in this study, and thus there is a chance that some unfortunate breeding crosses were made on heifers, causing worse dystocia (26). Research into risk factors for on-farm mortality has also shown that management before, and during calving, can have a significant effect on the mortality rate of cows (4). Further, it is known that the productive life of Norwegian beef cattle is too low with a third of Norwegian beef heifers only calving once (27). The reasons for this are complex and in part due to the structure of agricultural subsidies in Norway, combined with a “young cow” premium price at slaughter that can decrease “the financial incentive to increase lifetime calf production” (27). However, there is also a need for improved education amongst beef farmers and their advisors with the goal of improving beef cow management (27). Many of the certificates which listed palsy as the reason for OFES, also noted that the palsy was related to calving, meaning even more animals than the 17% reported in Table 3, underwent OFES because of obstetrical reasons. A higher proportion of cows calving for the first and second time were categorized to be slaughtered for obstetric reasons, than those in their second parity or older. This aligns with earlier research on dystocia, finding dystocia to be more common in heifers than in multiparous cows, and delivery to be more painful and stressful (28). Needing assistance or experiencing dystocia has also been found to be a risk factor for beef cows in Norway having fewer calves (27). It is unsurprising that obstetrical problems, recumbency and accidents of different kinds are a big part of the reasons for OFES, as the same has been found in on-farm mortality research (20, 29).

There is considerable focus on how to reduce the environmental impact of cattle herds and in doing so improving the sustainability of cattle production systems (30). Preventing acute injuries and disease or making use of OFES can reduce the waste of the animal. By reducing on-farm mortality, where no meat goes to human consumption, OFES can thus reduce the number of animals needed for the same yield. Dystocia is an important reason for acute suffering and sometimes leads to conditions or trauma that come with a poor prognosis (28). OFES can offer a solution to these cases of dystocia and prolapse, and thus salvage the meat, whereas trying treatment could yet end in euthanasia and destruction. However, there are important concerns surrounding OFES performed because of an obstetrical reason. Many of the cows with obstetrical causes of OFES are likely to be suffering acutely. Therefore, it is questionable to have them wait long for OFES. In that concern, further work is needed to analyze the wait time from accident to slaughter, or certification to slaughter. This article has identified animal welfare concerns in cattle production in Norway within the reasons for OFES, a subgroup of on farm mortality. It is important to prevent a high mortality rate in young animals to increase efficiency in cattle production in Norway. It can be theorized that the current housing of young animals in pens, where they have little option of escape, stimuli or motion, can increase the occurrence of trauma. However, further research is necessary to confirm or disprove this hypothesis. The obstetrical reasons for OFES are often preventable, either by careful breeding management and sire selection, or by calving management. Further, veterinarians choosing to reponate prolapses or preform c-section instead of choosing OFES could potentially lower mortality rates. A closer look into the overall management and treatment protocols within cattle health in Norway is therefore recommended based on the results of this study.

Animals undergoing OFES, are often recumbent and therefore are dirtier than normal slaughter cattle (31). This causes difficulty in maintaining good slaughter hygiene, risking cross-contamination from the skin to meat (13). This was a concern raised by some veterinarians from focus groups and interviews made in British Columbia (12). Additionally, 17% of the official veterinarians questioned in an Irish study perceived there to be a greater risk to consumers from consuming OFES meat (6). As 69% of the study sample in this study was categorized as either being slaughtered for recumbency conditions or locomotory conditions, higher contamination risk is probable on these animals, as their conditions would have them lie for longer than healthy animals. When evaluating OFES and trying to improve its practice, these public health concerns should be researched further.

Every third month was chosen for data gathering, over 1 year, 2018, to include 1 month in each season. The only significant difference in proportion between the fourth months (January-April-July-October), was within the beef production group. This was a difference between January and April, seen by fewer beef animals OFES for locomotory reasons in January than any other month meanwhile more animals OFES for recumbency and obstetric reasons in April than any other month. This could be explained by the seasonal differences in beef production where most beef herds in Norway have a spring calving season (32). It is, therefore, reasonable to assume that there would not be too big a difference in results if the study had included all 12 months in a year. The four slaughterhouses were chosen because of either size or their geographical placement in cattle-dense areas. They are found in three different regions of Norway, and therefore display practice in a big part of Norway. However, it could be that less OFES is practiced in fewer cattle-dense areas, although unlikely to have an effect overall, as it is offered in all areas in Norway. Nonetheless, for more acute reasons, such as obstetrics, or other painful conditions, a farmer or veterinarian could choose euthanasia instead of OFES if the distance to the slaughterhouse is great.

The validity of the veterinary certificates can be questioned, both regarding correctness and completeness (33). They are handwritten and some were filled out with a specific history of how the animal came to be OFES, others gave limited information, e.g., does not stand up, lame or palsy. Three of the categories reported reasons not eligible for OFES in Norway (9). These were management, where it was stated that the farmer asked for OFES, and then poor appetite and OFES for clinical internal signs of illness. The clear difference in how veterinarians filled out the certificate raised questions on how good the certification is, which then leads to questions on the food safety of the practice. At the time of data collection, there was no specific training for this for the private veterinarian. However, the new guidelines published in September 2022, demand that official veterinarians do the *antemortem* inspection and certification (9). A continuing education course is launching soon for private veterinarians to become official veterinarians for these tasks.

Although only four certificates were marked to have an illegible reason for OFES, many more were hard to read and missing information. The correctness of the certificates is thus compromised, by the human influence of what both farmer and veterinarian decide to put on the certificate (33). Additionally, the identification number was in some not correct, not written out in full, or handwriting hard to interpret, leading to uncertainty in food-chain information, another public health concern. Further, a comparison with reports from NDHRS, shows that the following culling reasons for OFES cows were most noted by farmers; 29.6% had an accident in the barn, 19.6% with other disease, 13.5% with calving problems and 12.4% with milk fever (34), and these proportions do not quite match the proportions in this study. The NDHRS records are hard to compare to this study, but the use of “disease” and milk fever as categories complicates this further, as this shouldn't fall under OFES. Comparing the certificates with *postmortem* findings would be a field of further research, but *postmortem* findings are mostly poorly documented if the carcass is not condemned. Digital veterinary certificates, with additional information on *postmortem* findings, could improve data collection, both for food chain information, as well as for further research. The Animal Health Portal (DHP) in Norway, an animal registry for animal health, artificial insemination, and food-chain information by Animalia, is already used by veterinarians and farmers and would be the most obvious place to link the certificate to Animalia (35).

The occurrence of OFES is uniquely high in Norway compared to other reported countries (6, 7). The reasons for this are complex. However, there is a long tradition for OFES in Norway, which is reflected in the interpretation and practicing of the regulatory guidelines, further the service is available to farmers all year-round, 24/7 (4). In contrast, OFES is only offered in limited areas, during specific periods, or not offered at all, in other countries where the legislation allows for OFES (1, 6, 20). Countries close by, like Sweden, Finland and Iceland make do with casualty slaughter and home slaughter for their own use, when deciding options for compromised slaughter-ready animals (4). The study results are therefore quite representative of the situation of OFES in Norway and may represent similar situations within other on-farm mortality options internationally. The availability of OFES in Norway may contribute to farmer and veterinarian making faster decisions in cases of acute trauma, leading to the animal's pain being alleviated soon, yet reducing the loss of income for the farmer. However, the timeframe from accident to alleviation may not be too long, and thus, further research is needed into how fast the process surrounding OFES works. The findings of such research may then answer quite a few raised concerns about animal welfare within OFES.

## Conclusion

This study is the first to report on the reasons for OFES in Europe. Almost half of the OFES cases were slaughtered for locomotion reasons, almost a quarter for recumbency and one of six for obstetric reasons. There were considerable differences in reasons for OFES between production systems and animal age groups. The results point to areas of improvement within both housing and management of cattle, through proactive culling plans and guidelines. The current system of certificates leaves some room for bias, both in their validity and in the insurance of public health, and therefore, digitalization of certification is recommended. Digitalization can further help contribute to animal health registries, animal welfare programs and epidemiology.

## Data availability statement

The raw data supporting the conclusions of this article will be made available by the authors, without undue reservation.

## Author contributions

GS were responsible for data collection. GS and IH-H were responsible for data analysis. GS, IH-H, and AM contributed to the writing of the article. All authors participated in the design of the study, edited, and approved the final manuscript.

## Funding

This project was funded by the Norwegian University of Life Sciences as a part of a research track student project.

## Conflict of interest

Author GM was employed by Norwegian Food Safety Authority. The remaining authors declare that the research was conducted in the absence of any commercial or financial relationships that could be construed as a potential conflict of interest.

## Publisher's note

All claims expressed in this article are solely those of the authors and do not necessarily represent those of their affiliated organizations, or those of the publisher, the editors and the reviewers. Any product that may be evaluated in this article, or claim that may be made by its manufacturer, is not guaranteed or endorsed by the publisher.

## Abbreviations

On-farm emergency slaughter
NDHRS: Norwegian Dairy Herd Recording System
NBCRS: Norwegian Beef Cattle Recording System
EU: European Union
EEA: European Economic Area.
